# Investigating the Neural Correlates of Emotion–Cognition Interaction Using an Affective Stroop Task

**DOI:** 10.3389/fpsyg.2017.01489

**Published:** 2017-09-01

**Authors:** Nora M. Raschle, Lynn V. Fehlbaum, Willeke M. Menks, Felix Euler, Philipp Sterzer, Christina Stadler

**Affiliations:** ^1^Department of Child and Adolescent Psychiatry, Psychiatric University Clinics, University of Basel Basel, Switzerland; ^2^Department of Psychiatry and Psychotherapy, Charité Universitätsmedizin Berlin Berlin, Germany

**Keywords:** emotion processing, emotion–cognition interaction, cognition, fMRI, Stroop

## Abstract

The human brain has the capacity to integrate various sources of information and continuously adapts our behavior according to situational needs in order to allow a healthy functioning. Emotion–cognition interactions are a key example for such integrative processing. However, the neuronal correlates investigating the effects of emotion on cognition remain to be explored and replication studies are needed. Previous neuroimaging studies have indicated an involvement of emotion and cognition related brain structures including parietal and prefrontal cortices and limbic brain regions. Here, we employed whole brain event-related functional magnetic resonance imaging (fMRI) during an affective number Stroop task and aimed at replicating previous findings using an adaptation of an existing task design in 30 healthy young adults. The Stroop task is an indicator of cognitive control and enables the quantification of interference in relation to variations in cognitive load. By the use of emotional primes (negative/neutral) prior to Stroop task performance, an emotional variation is added as well. Behavioral in-scanner data showed that negative primes delayed and disrupted cognitive processing. Trials with high cognitive demand furthermore negatively influenced cognitive control mechanisms. Neuronally, the emotional primes consistently activated emotion-related brain regions (e.g., amygdala, insula, and prefrontal brain regions) while Stroop task performance lead to activations in cognition networks of the brain (prefrontal cortices, superior temporal lobe, and insula). When assessing the effect of emotion on cognition, increased cognitive demand led to decreases in neural activation in response to emotional stimuli (negative > neutral) within prefrontal cortex, amygdala, and insular cortex. Overall, these results suggest that emotional primes significantly impact cognitive performance and increasing cognitive demand leads to reduced neuronal activation in emotion related brain regions, and therefore support previous findings investigating emotion–cognition interaction in healthy adults. Moreover, emotion and cognition seem to be tightly related to each other, as indicated by shared neural networks involved in both of these processes. Emotion processing, cognitive control, and their interaction are crucial for healthy functioning and a lack thereof is related to psychiatric disorders such as, disruptive behavior disorders. Future studies may investigate the neural characteristics of children and adolescents with disruptive behavior disorders.

## Introduction

An adequate handling of emotional information is a key factor for healthy functioning within our everyday life. How a person processes and regulates emotions impacts their cognition, behavior, and well-being (Dolan, 2002; Gross, 2002; John and Gross, 2004). Thereby, emotion processing not only influences cognitive control, but cognitive control may likewise affect emotions. Research has indicated that a fine balance of the emotion and cognition networks ultimately allows appropriate functioning (Hart et al., 2010). A failure to successfully process or regulate emotions is characteristic for different mental health disorders, including disruptive behavior disorders (Sterzer et al., 2005), attention-deficit/hyperactivity disorder (ADHD; Walcott and Landau, 2004), or psychosis (Livingstone et al., 2009). Therefore, an improved understanding of the mechanism supporting successful emotion regulation skills is of utmost personal, clinical, and societal relevance (Gross, 2002).

Behavioral research studies have demonstrated that emotional stimuli can positively or negatively impact cognitive processing. For example, the presentation of emotional stimuli has shown to disrupt working memory performance (Dolcos and McCarthy, 2006) and impact reaction times during a perceptual task (Gupta and Deak, 2015). Similarly, it was demonstrated that the presence of an emotional stimulus can reduce task accuracy and reaction times during Stroop task performance, which reflects cognitive control mechanisms (Blair et al., 2007; Hart et al., 2010; Uher et al., 2014). Visually presented and/or auditory-induced emotions can also positively influence cognition, resulting in improved accuracy or shorter reaction times during tasks including conflict processing, visual attention, or decision making (Schupp et al., 2007; Kanske and Kotz, 2011; Zinchenko et al., 2015). Factors that are known to influence the interaction of cognitive and emotional processes include cognitive load, level of threat, physical stimulus properties, position of emotional distractors (left or right hemifield), individual differences, and the availability of conflict-resolving brain resources (Arnsten and Goldman-Rakic, 1998; Hartikainen et al., 2000; Pessoa, 2009; Thompson et al., 2010; Cohen and Henik, 2012; Gupta and Raymond, 2012; Kanske, 2012; Okon-Singer et al., 2013; Gupta et al., 2016). By transiently enhancing or diminishing cognitive functioning, emotional states may thus impact the control of thoughts and behavior in order to meet situational demands (Gray et al., 2002).

Neuroimaging methods, such as functional magnetic resonance imaging (fMRI), can investigate the neural networks underlying emotional and cognitive processes as well as their interaction. Brain regions responsible for simple emotion-processing tasks are the amygdala, right insula, as well as the medial and ventrolateral prefrontal cortex (Phan et al., 2002; Dolcos and McCarthy, 2006; Van Dillen et al., 2009). Thereby, the engagement of individual brain regions depends on the quality of the emotion being processed. For example fear is particularly known to elicit amygdala activation, sadness is commonly represented by subcallosal cingulate activity, and emotion processing tasks with an additional cognitive component (e.g., emotional recall) also target the insular and anterior cingulate cortex (for a review see Phan et al., 2002). Brain regions associated with simple cognitive control (e.g., during working memory, conflict resolution, inhibition, or emotion regulation tasks) include the ventromedial, right (dorso-)lateral and orbital prefrontal cortex, lateral and right superior parietal cortex, and anterior cingulate cortex (Phan et al., 2002; Ochsner et al., 2004; Ochsner and Gross, 2005; Dolcos and McCarthy, 2006; Van Dillen et al., 2009; Pitskel et al., 2011).

To date, several fMRI studies have aimed at targeting the more complex interaction between cognition and emotion. The most commonly identified neural correlates of emotion–cognition interaction sites include parietal and prefrontal cortices, as well as limbic brain regions (i.e., cingulate, amygdala, and insula; Gray et al., 2002; Etkin et al., 2006; Blair et al., 2007; Van Dillen et al., 2009; Hart et al., 2010; Melcher et al., 2011; Kellermann et al., 2012; Gu et al., 2013; Cromheeke and Mueller, 2014). For example, Etkin et al. (2006) used an emotional conflict task and found that neural activation within the amygdala, dorsomedial-, and dorsolateral prefrontal cortex represents the level of emotional conflict, while the rostral anterior cingulate may reflect emotional conflict *per se* (Etkin et al., 2006). Likewise, Gu et al. (2013) identified shared and distinct brain regions responsible for cognitive and emotion processing or the interaction of both (Gu et al., 2013). In particular, an interaction effect was observed within in bilateral anterior insula, somatosensory cortices, and frontoparietal regions. Using an emotional working memory task, Gray et al. (2002) pinpointed left and right lateral prefrontal cortex as the site of emotion–cognition interaction. And finally, Blair et al. (2007) as well as Hart et al. (2010) combined emotional stimuli and Stroop task performance within their designs in order to elicit areas that are dynamically modulated either by increased emotional or enhanced cognitive demands. Again, bilateral amygdala, inferior frontal/ventrolateral prefrontal, and the cingulate cortex were identified as areas of neural changes dependent on cognitive and/or emotional load (Blair et al., 2007; Hart et al., 2010).

For the present study we adapted and re-evaluated the affective number Stroop task as implemented by Hart et al. (2010). Our goal was the investigation of dynamic changes in either the emotion or cognition network elicited by both variations in emotional content (through the use of negative as opposed to neutral images), and changes in cognitive demand (using different conditions of a number Stroop task). A further motivation for this study was the characterization of the neural correlates representing the effect of emotions on cognition in young adults as a basis for future studies in children and adolescents with social disorders (e.g., disruptive behavior disorders). This is of particular interest since behavioral studies have already demonstrated altered emotion–cognition interactions in disruptive behavior disorders (Euler et al., 2014). Nevertheless, the neural correlates in these clinical populations are still unknown. Therefore, our aims were to: (I) elicit activation in emotion-related brain regions in response to the affective primes implemented within our task (e.g., amygdala, insula, and prefrontal cortex Phan et al., 2002); (II) demonstrate activation in cognitive brain regions in response to the Stroop task (e.g., prefrontal cortex, lateral, and right superior parietal cortex, anterior cingulate cortex; Laird et al., 2005); (III) investigate previously identified brain regions that are significant in relation to the emotion–cognition interaction (e.g., amygdala, prefrontal cortex, and anterior insula; Gray et al., 2002; Etkin et al., 2006; Blair et al., 2007; Hart et al., 2010; Gu et al., 2013) and assess their involvement within the task described here. Based on strong prior behavioral evidence (Homack and Riccio, 2004), we hypothesized to observe delayed reaction times and reduced task accuracy for trials with increased cognitive load (i.e., from congruent to stars to incongruent Stroop trials), and for trials following affective (negative) primes compared to neutral primes. Neurally, we expected to replicate the above mentioned findings of changes in neural activation patterns within the emotion and/or cognition network in dependence to cognitive load (Gray et al., 2002; Etkin et al., 2006; Blair et al., 2007; Van Dillen et al., 2009; Hart et al., 2010; Melcher et al., 2011; Kellermann et al., 2012; Gu et al., 2013; Cromheeke and Mueller, 2014).

## Materials and methods

### Participants

Thirty healthy, German-speaking volunteers (mean age: 21.74 years; range 19–24 years; 15 males) with no prior psychological or neurological history were included in the current study. Participants took part in one testing session that included psychometric testing, one functional neuroimaging task and a T1-weighted structural image acquisition. Two participants were excluded from analysis since one of them had completely missing and the other person very low in-scanner performance (e.g., more than 20% misses in each run). All participants were further right-handed, had normal or corrected-to-normal vision, and provided written informed consent as approved by the local ethics committee (Ethikkommission der Nordwest- und Zentralschweiz).

### Psychometrics

Participants included in this study completed a battery of standardized tests comprising verbal and non-verbal IQ [German version of the Vocabulary and Matrix reasoning subtests of the WAIS-IV (Petermann, 2012), present mood (EWL; Janke, 1978)], behavioral and emotional functioning (YSR; Achenbach, 1991), psychopathic traits (YPI; Andershed et al., 2007), and handedness (EDI; Caplan and Mendoza, 2011). The YPI, YSR, and EWL were missing for one person. The resulting behavioral group characteristics are provided in Table 1.

**Table 1:** Behavioral group characteristics.

		**Mean ± *SD***
**Age** (in years)		21.73 ± 1.53
**IQ (WAIS-IV)**	Vocabulary	12.63 ± 3.15
	Matrix reasoning	10.83 ± 1.37
**YPI**	Dishonest charm	9.31 ± 2.88
	Grandiosity	8.55 ± 2.95
	Lying	7.10 ± 1.40
	Manipulation	7.76 ± 2.20
	Remorselessness	7.45 ± 2.25
	Unemotionality	10.52 ± 3.19
	Callousness	12.28 ± 1.65
	Thrill-seeking	12.31 ± 2.65
	Impulsiveness	11.14 ± 2.77
	Irresponsibility	8.28 ± 2.63
	Grandiose manipulative dimension	8.18 ± 1.84
	Callous unemotional dimension	10.08 ± 1.51
	Impulsive irresponsible dimension	10.57 ± 2.04
	Total score	9.56 ± 1.41
**YSR**	Withdrawn	56.66 ± 7.34
	Somatic complaints	54.55 ± 5.23
	Anxiety/Depression	54.86 ± 5.15
	Social problems	53.34 ± 4.41
	Thought problems	52.76 ± 4.94
	Attention problems	55.28 ± 5.94
	Delinquent behavior	54.03 ± 5.74
	Aggressive behavior	52.14 ± 3.98
	Total score internalizing behavior	53.52 ± 8.03
	Total score externalizing behavior	50.03 ± 6.90
	Total score problem scale	52.69 ± 7.95

*For WAIS-IV, standard scores are reported; for YPI, mean scores are reported; and for YSR, t-values are reported*.

### fMRI—task procedure

**Figure 1:**
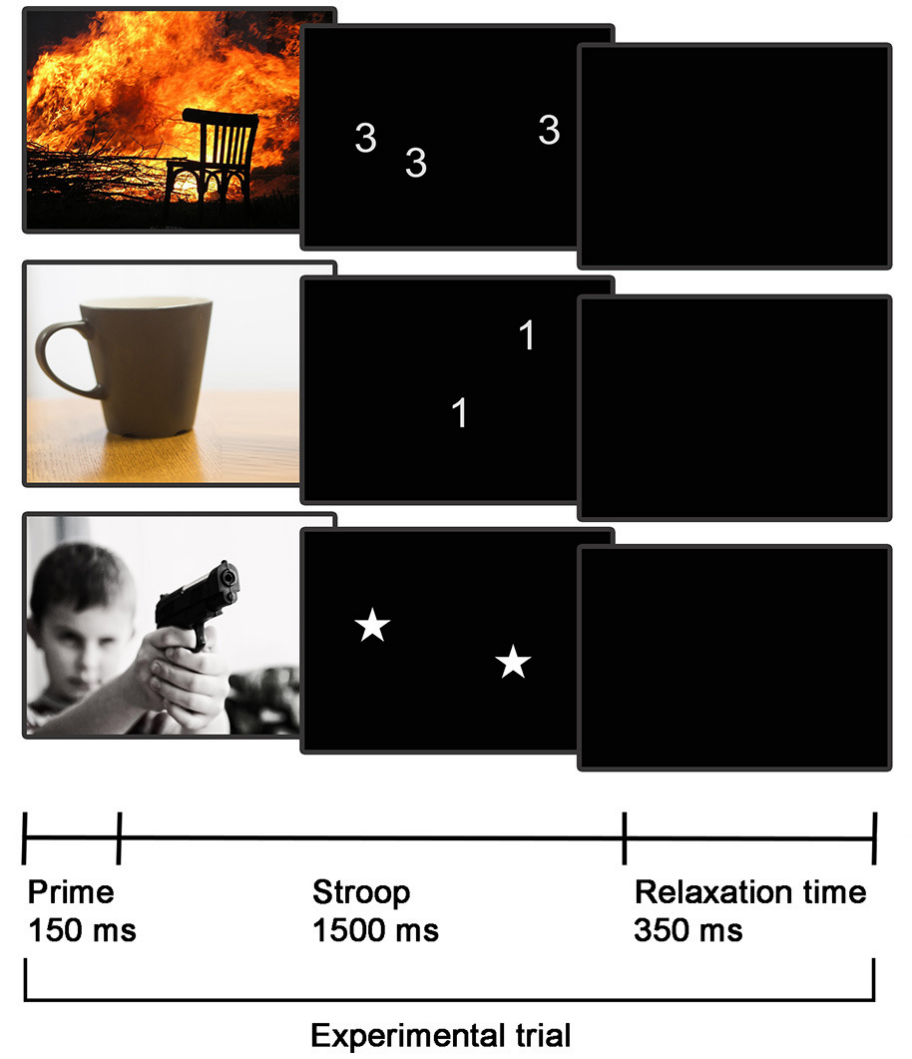
fMRI task design. Three exemplary emotional Stroop trials are displayed, depicting the following conditions (from top to bottom): Negative-congruent trial, neutral-incongruent trial, and negative-stars trial.

The neuroimaging session included event-related functional neuroimaging during the performance of an emotional number Stroop task. Additionally, T1-weighted structural images were acquired for each participant. The emotional number Stroop task was adapted and modified based on a design described by Hart et al. (2010) (see trial design in Figure 1). We decided to use a number Stroop task as it has particularly been developed for use in the MR environment and has previously successfully been implemented in neuroimaging research studies (e.g., Blair et al., 2007; Hart et al., 2010). Each trial started with an emotional prime of either neutral or negative valence, presented for 150 ms. Negative (Neg) or neutral (Neu) primes were first followed by an item of the number Stroop task presented for 1,500 ms, before a short relaxation period of 350 ms ended the trial. During the number Stroop task, participants were presented with an array of 1, 2, 3, or 4 digits and were asked to indicate through button press the number of items presented. The number of items was either congruent (C) in relation to the printed digits (e.g., the digit 4 in an array of 4) or incongruent (IC) with the printed digits (e.g., digit 4 in an array of 3). Star shaped stimuli (S) were used as a control condition (no interference of digit and item number) and null trials (trials with a black screen instead of the Stroop trial) were added during the randomization process. Emotional stimuli were adapted from the Developmental Affective Photo System (DAPS; a child-appropriate picture system Cordon et al., 2013), which uses part of the IAPS (International Affective Picture System Lang et al., 2008) commonly used in adults. We implemented DAPS images because this task was designed to ultimately be employed in children and adolescents with psychiatric disorders. However, given that all images remained part of the IAPS system, the chosen stimuli were considered suitable for both adult and adolescent populations. A list of the images used is provided in Supplemental Information 1. In combination with the negative or neutral primes, the following combinations of prime and trial condition were possible: negative-congruent (Neg_C), negative-stars (Neg_S), negative-incongruent (Neg_IC), and neutral-congruent (Neu_C), neutral-stars (Neu_S), neutral-incongruent (Neu_IC). Prior to study start, our task was behaviorally tested in adults. Pilot data assessment indicated a significant emotion by cognition interaction for reaction time and accuracy measurements (see Supplemental Information 1.2).

Prior to the start of the experiments, an optimal stochastic trial order allowing for a rapid event-related design was determined using optseq2, a tool for automatically scheduling events for rapid-presentation event-related fMRI experiments (for further information see http://surfer.nmr.mgh.harvard.edu/optseq/ or experiments using similar designs Ferri et al., 2012; Kuhlmann et al., 2016). We administered a total of 300 Stroop trials (100 for each C/S/IC) and 50 null trials. All 300 Stroop trials were preceded by either neutral or negative primes (50:50). Total scan time was about 11.5 min. The complete experiment was performed over the course of 2 runs. At the end of the neuroimaging session, participants were further asked to perform valence ratings of the images presented in the scanner using a Likert scale from −2 to 2 (with −2 representing very negative valence, 0 being neutral, and 2 indicating high attractiveness of the stimulus). For each participant the mean scores of the negative images affect rating were used as a covariate of no interest within the group analysis to account for differences in valence judgments between the young adults (see also Supplemental Information 2.1 Emotional Valence Rating).

### fMRI—image acquisition and analysis

Whole brain blood oxygen level-dependent (BOLD) fMRI data and T1-weighted mprage images were acquired on a Siemens 3T MR imaging system (Siemens Prisma, Erlangen, Germany) and a 20-channel phased-array radio frequency head coil. For the fMRI task a rapid event-related stochastic design with TR = 2,000 ms, TE = 30.0 ms, FOV = 192 mm; image matrix = 64 × 64 mm; voxel size = 3 mm and number of slices = 37 was used. We further acquired a high resolution T1-weighted structural image using the following specifications: TR = 1,900.0 ms; TE = 3.42 ms; FOV = 256; image matrix = 256 × 256; voxel size = 1 mm. T1-weighted mprage structural neuroimaging data was used for co-registration and to calculate the total intracranial volume (TIV). Men and women are known to differ in overall brain size (Leonard et al., 2008; Luders et al., 2009; Giedd et al., 2012). This was also true for the present sample where TIV significantly differed between males and females [males *M* = 1,529.52 ± 87.22/females *M* = 1,360.72 ± 91.99; *t*_(28)_ = 5.16, *p* < 0.001]. Likewise, socioeconomic status, sex, and age all correlate with TIV (Luders et al., 2009, 2014; Taki et al., 2011; Jednorog et al., 2012). This was accounted for by using a covariate of no interest in consequent random effects analyses. Therefore, TIV was extracted through the voxel-based morphometry toolbox (VBM8; http://dbm.neuro.uni-jena.de/vbm) as implemented in SPM8 and executed in MATLAB (Mathworks, Natick, MA).

All functional MRI data was analyzed using SPM8 (http://www.fil.ion.ucl.ac.uk/spm/). Preprocessing included slice timing correction, realignment, co-registration to the structural images, segmentation of the structural image, normalization to the Montreal Neurologic Institute (MNI) standard brain, and smoothing using an 8 mm full-width at half maximum Gaussian kernel. During single subject analysis, the following regressors were built: Neg_C, Neg_S, Neg_IC, Neu_C, Neu_S, Neu_IC. Contrast images were created to investigate (1) the main effect of emotion (Neg>Neu trials), (2) the main effect of cognition (IC>C or IC>S trials), and (3) the influence of emotion on cognition along with increasing cognitive demand as based on two-sample *t*-tests comparing Neg_C vs. Neu_C, Neg_S vs. Neu_S, and Neg_IC vs. Neu_IC trials.

Due to the challenges in capturing the intricate nature of emotion–cognition interactions, the majority of publications in this field have based their interpretation on a priori based regions of interests only. Here, we present both small volume peak-level FWE-corrected findings at *p* < 0.05 for the main regions of interest [i.e., amygdala, insula, and/inferior frontal junction/precentral gyrus according to previous literature (Gray et al., 2002; Etkin et al., 2006; Blair et al., 2007; Hart et al., 2010; Gu et al., 2013); defined anatomically using the automated anatomical labeling atlas (Tzourio-Mazoyer et al., 2002)] and uncorrected, exploratory, whole brain findings (*p* < 0.001).

### Region of interest analyses

In order to further characterize the effects of cognitive load on the neural basis of emotion–cognition interactions, we further extracted mean peak activation scores as based on FWE-corrected findings from the two main contrasts targeting the emotion (Neg>Neu trials) and cognition (IC>S trials) networks of the brain. More specifically the signal change at the local peak activation scores for bilateral amygdala, right insula, and bilateral precentral gyrus were extracted using the marsbar toolbox (Brett et al., 2002) and further assessed using paired-samples *t*-tests.

### In-scanner performance

In-scanner performance was assessed by computing the mean accuracy and reaction time in response to Neg_C, Neg_S, Neg_IC, Neu_C, Neu_S, and Neu_IC Stroop trials. For both accuracy and reaction times two separate 2 (emotion: Neg, Neu) by 3 (task: C, S, IC) repeated measures ANOVAs were performed in order to investigate the main effect of task, main effect of emotion, and the influence of emotion on task.

## Results

### In-scanner performance

The 2 (emotion: Neg, Neu) by 3 (task: C, S, IC) ANOVA on *task accuracy* (i.e., correctly answered Stroop trials) indicated a significant main effect of emotion [*F*_(1, 29)_ = 7.34, *p* = 0.011] and a main effect of cognition [*F*_(2, 28)_ = 12.38, *p* < 0.0001]. Bonferroni corrected *post-hoc* tests indicated that the main effect on emotion was constituted by lower accuracy following negative primes (compared to neutral primes) during Stroop task. Furthermore, significant differences in accuracy derived from the incongruent condition compared to the congruent (*p* < 0.0001) and stars condition (*p* = 0.002). However, the difference between congruent and stars conditions did not reach significance (*p* = 1.00). Finally, the emotion by cognition interaction did not reach significance [*F*_(2, 28)_ = 0.33, *p* = 0.722].

The 2 × 3 ANOVA implementing *reaction time* revealed a main effect of emotion [*F*_(1, 29)_ = 11.93, *p* = 0.002] and a main effect of cognition [*F*_(2, 28)_ = 80.27, *p* < 0.001]. Bonferroni corrected *post-hoc* tests revealed significant reaction time differences for incongruent compared to congruent (*p* < 0.0001), congruent compared to stars (*p* = 0.019), and incongruent compared to stars (*p* < 0.0001) conditions. The emotion by cognition interaction did not reach significance [*F*_(2, 28)_ = 0.54, *p* = 0.590]. An overview of the in-scanner performance as based on the mean accuracy and reaction time for the whole group is given in Table 2.

**Table 2:** In-scanner performance (accuracy, reaction times).

		**Congruent [±*SD*]**	**Stars [±*SD*]**	**Incongruent [±*SD*]**
Accuracy	Negative prime	49.2 [1.5]	49.3 [1.0]	47.7 [3.1]
[raw scores]	Neutral prime	49.7 [1.1]	49.6 [0.9]	48.3 [2.4]
Reaction times	Negative prime	720.5 [72.6]	731.0 [84.3]	800.0 [88.9]
[ms]	Neutral prime	704.5 [66.0]	718.6 [73.4]	791.9 [90.3]

### fMRI results

**Figure 2:**
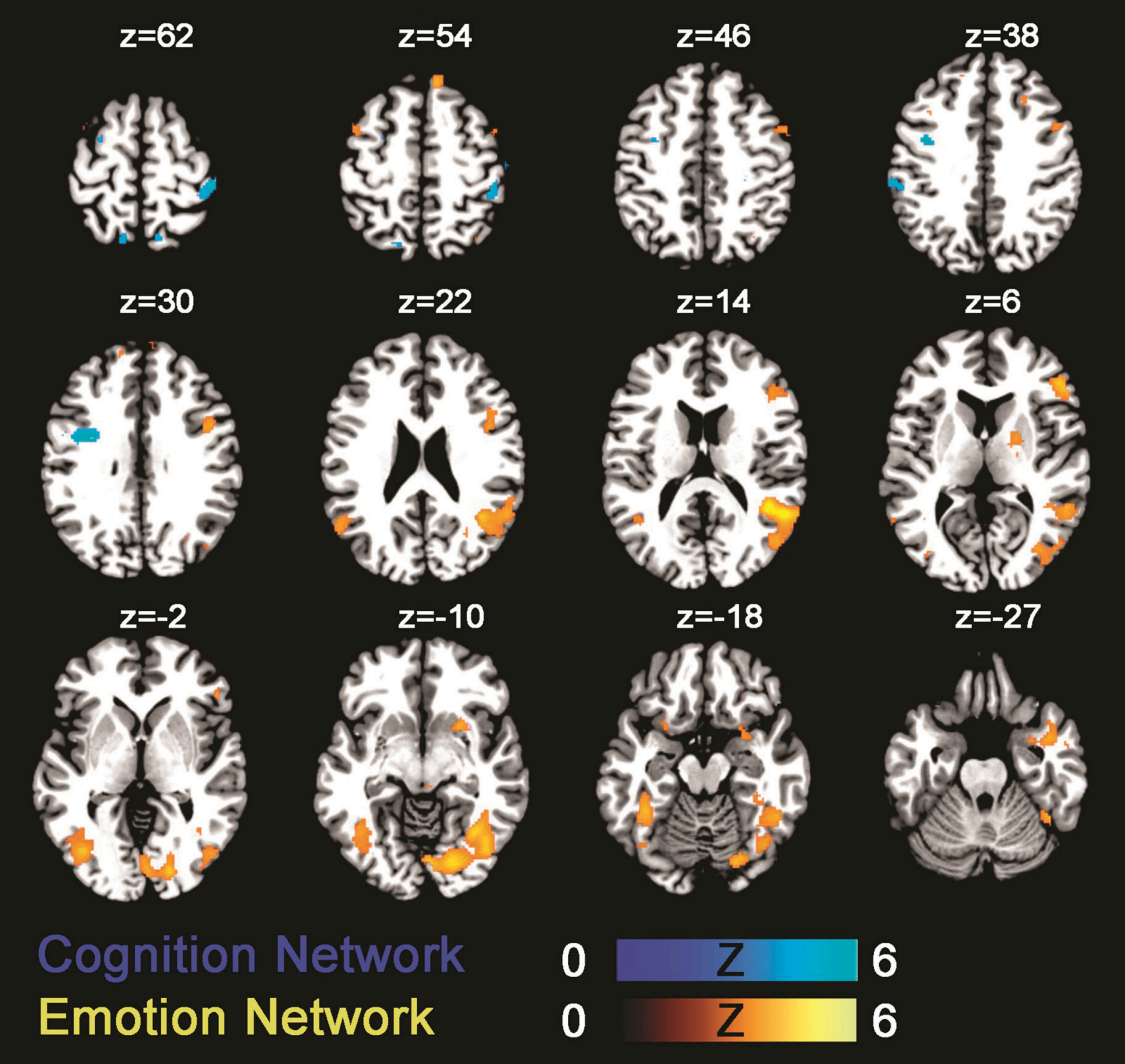
Statistical parametric maps showing brain activation linked to the emotion network (green-blue; negative > neutral trials) and the cognition network (gold-yellow; incongruent > congruent). Results are displayed at a *p* < 0.001, uncorrected threshold and neurologically displayed on axial slices using the Multi-image Analysis GUI as available at http://ric.uthscsa.edu/mango/mango.html.

The fMRI result part is organized in line with our a priori listed main aims: (1) testing the activation of the emotion network by use of the negative prime (Neg>Neu trials); (2) assessing the activation of the cognition network by comparing an incongruent to a neutral Stroop condition (IC>S); (3) evaluating the emotion–cognition interaction dependent on increased cognitive load (Neg_C vs. Neu_C, Neg_S vs. Neu_S, and Neg_IC vs. Neu_IC trials). First, testing the (1) emotion processing network revealed that trials with a preceding negative prime compared to those with a preceding neutral prime led to significant increases in activation in known emotion processing areas of the brain (Phan et al., 2002), including insula, amygdala, and prefrontal cortices (for an overview of activated areas see Table 3, Figure 2). Secondly, testing the **(2)** cognition network by use of the Stroop task (IC>S) revealed activations in areas including left precentral gyrus (FWE-corrected) and uncorrected within further areas including the superior frontal brain regions, temporal cortex, and insula (Table 3, Figure 2).

**Table 3:** MNI coordinates, cluster size, and Z-scores for significant FWE small-volume corrected results (indicated with bold letters) and uncorrected (*p* < 0.001; indicated with an asterix^*^) whole brain findings representing the emotion processing network (negative trials > neutral trials) and the cognition network (incongruent > stars trials) elicited by the given task.

**Brain region**	**Hem**	***k***	***Z*_0_**	**MNI coordinates (mm)**		
				**x**	**y**	**z**
**EMOTION NETWORK (NEG**>**NEU TRIALS)**						
**Precentral**	**R**	**187**	**3.84**	**46**	**6**	**30**
**Insula**	**R**	**29**	**4.67**	**26**	**8**	−**14**
**Amygdala**	**R**	**19**	**3.65**	**30**	**0**	−**26**
**Amygdala**	**L**	**1**	**3.16**	−**22**	**6**	−**18**
^*^Occipital, temporal lobe, including calcarine, fusiform, lingual, angular gyrus	R/L	3241	5.04	44	−48	12
^*^Inferior/middle temporal/occipital lobe, including fusiform gyrus	L	793	4.35	−38	−46	−16
^*^Inferior orbitofrontal lobe, including precentral gyrus	R	662	4.59	52	32	4
^*^Cerebellum	L	249	4.05	−12	−80	−40
^*^Middle temporal, superior marginal, angular gyrus	L	186	3.8	−52	−60	20
^*^Superior frontal lobe	R	73	4.15	10	38	54
^*^Cerebellum	L	72	3.93	−10	−50	−48
^*^Putamen, pallidum	R	68	3.89	20	−4	8
^*^Middle frontal lobe, precentral gyrus	L	58	3.55	−44	6	56
^*^Angular gyrus, superior parietal lobe	R	58	3.33	34	−64	50
^*^Middle temporal lobe	R	39	3.63	58	−2	−24
^*^Superior temporal pole, insula, olfactory, inferior/superior orbitofrontal	L	29	3.46	−24	10	−18
^*^Middle/superior occipital lobe	R	28	3.48	28	−66	24
^*^Superior medial frontal lobe	R	18	3.25	8	60	30
^*^Middle frontal gyrus	R	15	3.57	26	26	40
^*^Inferior/middle temporal lobe	L	13	3.28	−44	−8	−22
^*^Inferior frontal, pars opercularis, middle frontal lobe	L	12	3.61	−36	18	36
^*^Superior frontal lobe	L	10	3.32	−12	54	30
^*^Superior frontal lobe	L	9	3.38	−16	42	36
^*^Middle temporal gyrus	L	9	3.21	−60	−56	6
^*^Cerebellum	R	7	3.31	10	−32	−22
^*^Middle occipital lobe	R	7	3.18	42	−74	32
^*^Superior medial frontal, superior motor area	L	5	3.32	−4	28	66
^*^Caudate	L	3	3.14	−8	10	8
^*^Middle temporal pole	L	2	3.16	−40	16	−34
^*^Middle temporal pole	L	1	3.25	−38	14	−30
^*^Middle occipital lobe	L	1	3.14	−36	−66	16
^*^Superior temporal pole	L	1	3.12	−36	12	−28
^*^Thalamus	R	1	3.11	10	−6	0
**COGNITION NETWORK (IC**>**S)**						
**Precentral**	**L**	**87**	**4.03**	−**36**	−**2**	**34**
^*^Caudate	R	120	3.89	−4	10	16
^*^Paracentral lobule, supplementary motor area	R/L	96	3.7	−6	−30	64
^*^Superior temporal lobe	R	79	3.86	56	−30	14
^*^Superior temporal lobe, heschl's gyrus, insula, rolandic operculum	R	48	3.98	46	−18	6
^*^Superior temporal lobe	L	27	3.41	−58	−16	4
^*^Postcentral gyrus	R	20	3.58	52	−20	60
^*^Insula, rolandic operculum	L	15	3.8	−42	−10	18
^*^Putamen	L	15	3.51	−24	−6	18
^*^Superior frontal lobe	R	4	3.31	14	42	30
^*^Thalamus	L	2	3.2	−16	−22	16
^*^Superior temporal lobe	R	1	3.19	52	−26	10

*Results reported at small-volume FWE correction of p < 0.05*.

* *additional uncorrected whole brain clusters at p < 0.001*.

*Hem, Hemisphere; k, cluster size; Neg, negative; Neu, neutral; C, congruent condition; IC, incongruent condition; S, stars condition*.

**Figure 3:**
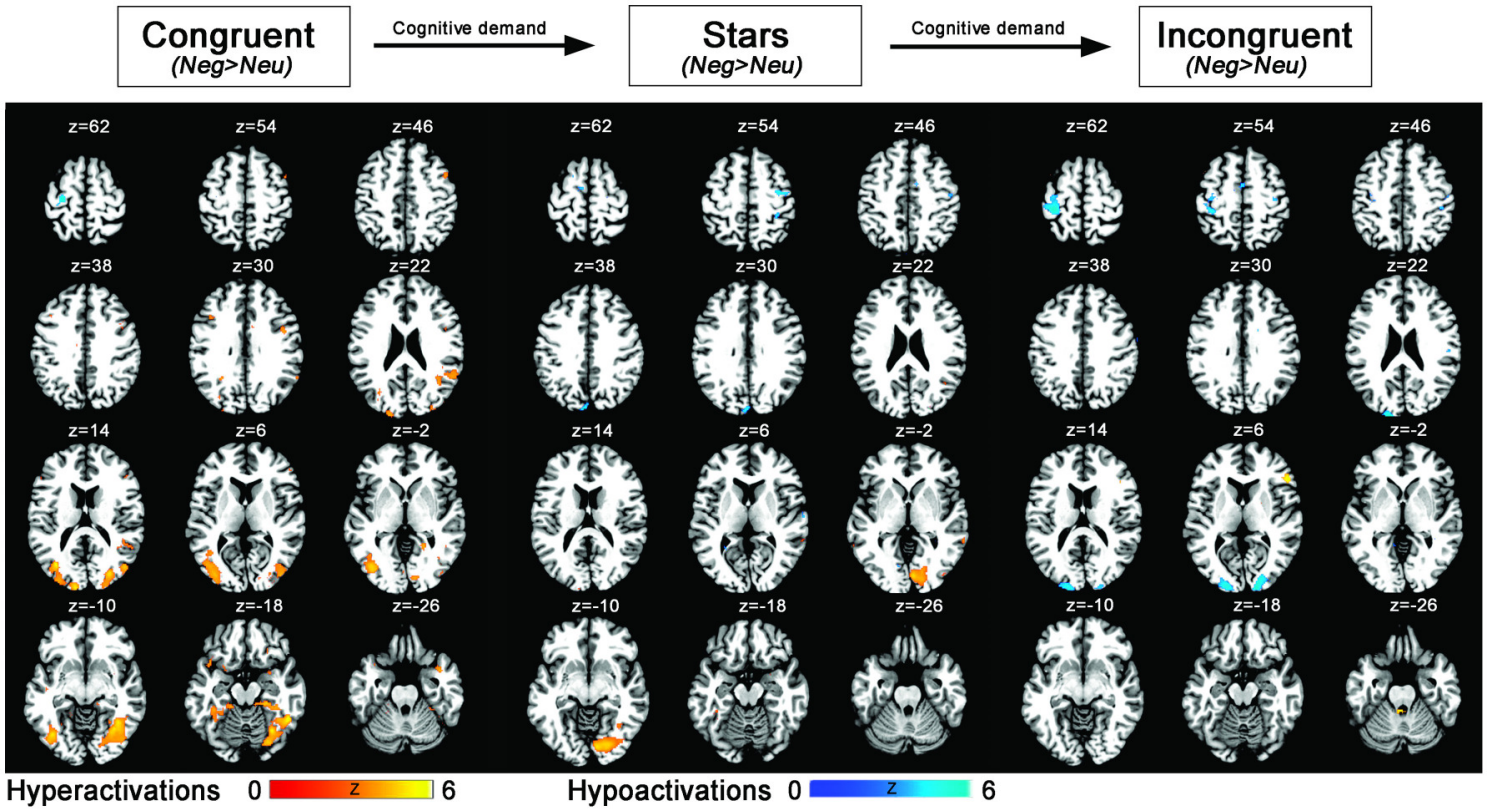
Statistical parametric maps showing emotion–cognition interaction related brain activation (blue: hypoactivations; red: hyperactivations) for negative > neutral emotional primes and the conditions congruent, stars, incongruent (ordered by lowest to highest cognitive load). Results are displayed at a *p* < 0.001, uncorrected threshold and neurologically displayed on axial slices using the Multi-image Analysis GUI as available at http://ric.uthscsa.edu/mango/mango.html.

Both control conditions (IC>S and IC>B) were contrasted with the IC condition. However, we decided to focus on IC>S trials for definition of the cognition network since more prefrontal activation was evoked, potentially due to different effects of the primes on congruent as opposed to stars trials. This procedure is in line with similar previous fMRI Stroop publications (for a review see Laird et al., 2005). Finally, (3) the influence of emotion on cognitive control was measured by contrasting Stroop trials with a prior negative prime to those Stroop trials following a neutral trial. Two-sample *t*-tests for negative vs. neutral trials were calculated for Stroop trials with increasing cognitive load (from congruent, to stars, and incongruent condition) and revealed differential activations for each of the three contrasts reflecting the influence of emotion on cognitive task performance as modulated by differential cognitive load. More specifically, for the contrast “Neg>Neu trials” during the congruent Stroop task condition, significant increases in activations were identified in bilateral amygdala, right insula, and right precentral gyrus (FWE-corrected) and on a whole-brain uncorrected level within further regions of the occipital, temporal, and inferior/middle frontal gyrus. For the opposite contrast “Neg < Neu trials” during the congruent Stroop trial, FWE-corrected activation within left precentral gyrus was observed. For the contrast “Neg>Neu trials” during the stars Stroop task condition, significant FWE-corrected findings were identified in right amygdala, while further cluster of activations were located within bilateral inferior occipital cortex (uncorrected whole brain approach). For the opposite contrast “Neg < Neu trials” significant activation was located within right precentral gyrus (FWE-corrected) and using a whole brain approach further clusters were located within occipital and superior frontal brain regions, left precuneus of the superior parietal lobe. Finally, for the contrast “Neg>Neu trials” during the incongruent Stroop task condition significant clusters of activations were based in inferior frontal and middle frontal brain regions (uncorrected findings only), while the opposite contrast of “Neg < Neu trials” during the incongruent Stroop task condition led to activity within left insula (FWE-corrected) as well as in further occipital brain regions, left pre- and postcentral gyrus, when using an uncorrected whole-brain approach (Table 4, Figure 3).

**Table 4:** MNI coordinates, cluster size, and Z-scores for significant FWE small-volume corrected results (indicated with bold letters) and uncorrected (*p* < 0.001; indicated with an asterix^*^) whole-brain findings representing the influence of emotion on cognitive processes dependent on cognitive load (i.e., congruent, stars, and incongruent trials).

**Brain region**	**Hem**	***k***	***Z*_0_**	**MNI coordinates (mm)**		
				***x***	***y***	***z***
**CONGRUENT (NEGC**>**NEUC)**						
**Precentral**	**R**	**64**	**3.79**	**46**	**0**	**32**
**Insula**	**R**	**7**	**3.65**	**30**	**8**	**−16**
**Amygdala**	**R**	**6**	**3.39**	**28**	**2**	**−16**
**Amygdala**	**L**	**2**	**3.33**	**−22**	**6**	**−18**
^*^Occipitotemporal including fusiform, calcarine, lingual gyrus, cuneus, cerebellum	R	2037	4.88	32	−66	−14
^*^Temporal/occipital lobe, including fusiform gyrus, cuneus	L	1470	4.64	−34	−72	14
^*^Middle/superior temporal lobe, angular gyrus	R	361	4.24	44	−42	18
^*^Inferior temporal lobe, cerebellum, fusiform, parahippocampal gyrus	L	303	3.95	−34	−52	−14
^*^Cerebellum, fusiform, lingual, parahippocampal gyrus	R	222	4.39	22	−34	−20
^*^Inferior/middle frontal lobe, including pars triangularis and opercularis	L	54	3.57	−40	18	32
^*^Superior temporal pole, inferior orbitofrontal lobe	L	51	3.77	−42	16	−16
^*^Amygdala	R	47	4	30	6	−14
^*^Lingual, parahippocampal gyrus	R	46	4.01	22	−50	−4
^*^Cerebellum	L	44	3.75	−10	−54	−50
^*^Superior temporal pole	R	30	3.82	40	6	−26
^*^Lingual gyrus	L	28	3.43	−16	−52	−6
^*^Inferior/superior orbitofrontal lobe	R	24	4.29	22	30	−16
^*^Middle cingulum	R	19	3.64	10	6	34
^*^Superior temporal pole, superior orbitofrontal lobe, insula	L	14	3.36	−26	12	−18
^*^Inferior frontal lobe, including pars triangularis	R	14	3.26	46	30	16
^*^Middle/superior temporal pole, inferior orbitofrontal lobe	L	12	3.53	−34	14	−24
^*^Inferior frontal lobe, including pars triangularis	R	12	3.35	40	24	18
^*^Middle temporal lobe	R	11	3.37	48	−50	0
^*^Middle/superior occipital lobe	L	10	3.29	−24	−92	32
^*^Cerebellum	R	9	3.32	32	−46	−44
^*^Inferior frontal gyrus, including pars opercularis	L	5	3.44	−36	6	24
^*^Middle/superior temporal lobe	L	5	3.27	−46	−16	−8
^*^Inferior/superior parietal lobe	L	5	3.22	−28	−54	48
^*^Inferior frontal lobe, including pars triangularis	R	5	3.19	54	38	6
^*^Middle cingulum	L	4	3.35	−10	−14	40
^*^Middle/superior occipital lobe	R	4	3.22	28	−72	36
^*^Inferior frontal lobe, including pars triangularis	L	4	3.17	−52	30	16
^*^Middle/superior occipital lobe	R	4	3.16	30	−72	26
^*^Cerebelum	R	4	3.13	40	−58	−32
^*^Supplementary motor area	R	2	3.3	10	8	72
^*^Lingual gyrus	L	2	3.16	−2	−80	−2
^*^Superior occipital lobe	L	1	3.14	−20	−66	38
^*^Angular gyrus	R	1	3.09	30	−60	52
**CONGRUENT (NEGC<NEUC)**						
**Precentral**	**L**	**100**	**4.21**	**−26**	**−20**	**60**
**STARS (NEGS>NEUS)**						
**Amygdala**	**R**	**1**	**3.66**	**26**	**2**	**−18**
^*^Inferior occipitotemporal, including lingual, fusiform, calcarine, cerebellum	R/L	862	5.27	8	−84	−6
^*^Inferior temporal lobe, including fusiform gyrus	L	52	3.56	−40	−42	−16
^*^Middle temporal lobe	R	50	4.11	66	−42	2
^*^Inferior temporal lobe, including fusiform gyrus	R	41	3.7	36	−58	−10
^*^Middle temporal lobe	R	40	4.01	56	0	−22
^*^Gyrus rectus, amygdala	R	19	3.65	20	10	−16
^*^Cerebellum	L	14	3.38	−14	−70	−30
^*^Inferior occipital lobe	R	8	3.3	42	−82	−4
^*^Middle temporal, lobe including angular gyrus	R	8	3.21	44	−60	20
^*^Middle/superior temporal pole	L	7	3.32	−38	12	−28
^*^Middle temporal lobe	L	5	3.3	−64	−50	0
^*^Superior occipital lobe	L	5	3.19	−12	−100	12
^*^Cerebelum	R	4	3.24	34	−66	−50
^*^Fusiform gyrus	R	3	3.24	42	−44	−14
^*^Superior occipital lobe	R	3	3.14	26	−84	24
^*^Angular gyrus	R	2	3.16	40	−72	50
^*^Cerebellum	R	2	3.15	12	−32	−20
^*^Putamen	R	1	3.2	26	14	−6
^*^Inferior occipital lobe	L	1	3.16	−40	−72	−6
^*^Angular gyrus	R	1	3.16	44	−70	50
^*^Anterior cingulate	R	1	3.15	14	40	16
^*^Inferior occipital lobe	L	1	3.12	−42	−68	−6
^*^Middle orbitofrontal lobe	R	1	3.14	26	38	−12
^*^Thalamus	L	1	3.1	−6	−14	2
^*^Superior temporal pole	R	1	3.1	40	4	−24
**STARS (NEGS<NEUS)**						
**Precentral**	**R**	**53**	**4.12**	**34**	**−12**	**56**
^*^Superior occipital lobe, including cuneus, precuneus	L	127	4.58	−6	−92	36
^*^Inferior parietal lobe, postcentral gyrus	R	31	3.61	32	−42	52
^*^Supplementary motor area	L	24	3.42	−10	−8	64
^*^Precuneus	L	16	3.63	−26	−52	4
^*^Middle cingulum, supplementary motor area	R	14	3.66	10	−2	44
^*^Lingual gyrus	L	14	3.22	−10	−72	−4
^*^superior parietal lobe, postcentral gyrus	R	12	3.23	14	−54	66
^*^Superior temporal lobe	R	11	3.33	66	−14	6
^*^Superior parietal lobe	R	6	3.24	24	−54	64
^*^Precentral gyrus	R	2	3.34	22	−18	62
^*^Superior frontal lobe	L	2	3.16	−18	6	62
**INCONGRUENT (NEGIC>NEUIC)**						
^*^Inferior frontal lobe, including pars triangularis	R	108	3.94	54	30	4
^*^Cerebellum, vermis	R/L	32	3.72	−2	−40	−28
^*^Middle frontal lobe	L	20	3.56	−40	10	60
^*^Pallidum, putamen	R	5	3.4	24	0	4
^*^Superior/medial frontal lobe	L	3	3.17	−6	36	58
^*^Caudate	L	1	3.11	−6	8	12
^*^Cerebellum	L	1	3.1	−10	−72	−36
**INCONGRUENT (NEGIC<NEUIC)**						
**Insula**	**L**	**14**	**3.67**	**−36**	**−2**	**10**
^*^Postcentral, precentral gyrus	L	614	4.35	−34	−32	62
^*^Middle/superior occipital lobe, including cuneus	L	426	4.24	−18	−98	20
^*^Middle/superior occipital lobe, including cuneus, calcarine gyrus	R	239	4.55	16	−100	8
^*^Postcentral, precentral gyurs	R	25	3.56	38	−22	52
^*^Supramarginal gyrus, postcentral	R	23	3.39	44	−30	48
^*^Supplementary motor area	R/L	22	3.29	−2	−4	54
^*^Postcentral, precentral gyrus	R	14	3.41	52	−18	42
^*^Supramarginal gyrus, rolandic operculum	R	10	3.3	54	−22	22
^*^Cerebellum, lingual gyrus	L	9	3.32	−10	−50	−2
^*^Postcentral gyrus	R	9	3.24	64	−6	38
^*^Precentral gyrus	L	9	3.18	−22	−14	72
^*^Superior occipital lobe, including cuneus	L	6	3.45	−8	−88	42
^*^Precentral gyrus	R	5	3.27	30	−16	70
^*^Supplementary motor area	L	4	3.33	−12	−14	50
^*^Precuneus	L	3	3.17	−12	−42	4
^*^Precentral gyrus	L	2	3.28	−52	0	24
^*^Postcentral gyrus	R	2	3.13	62	0	32
^*^Precentral gyrus	R	1	3.13	26	−28	76
^*^Precentral gyrus	R	1	3.12	64	2	28
^*^Lingual gyrus	L	1	3.11	−6	−56	0
^*^Postcentral gyrus	R	1	3.1	26	−46	64
^*^Supplementary motor area	L	1	3.1	−10	−10	54

*Results reported at small-volume FWE correction of p < 0.05*.

* *additional uncorrected whole brain clusters at p < 0.001*.

*Hem, Hemisphere; k, cluster size; Neg, negative; Neu, neutral; C, congruent condition; IC, incongruent condition; S, stars condition*.

### Region of interest analyses

#### Results

**Figure 4:**
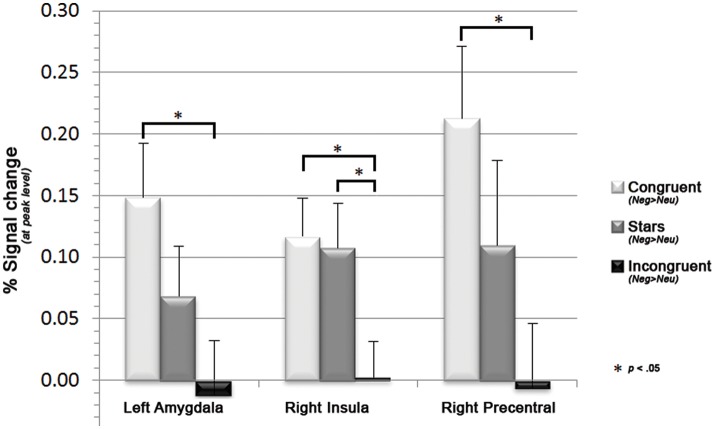
Bar graphs displaying decreases in the mean parameter estimates in left precentral gyrus and right amygdala along with increasing cognitive demand (i.e., for congruent, stars, and incongruent Stroop trials), as well as the associated sagittal brain slices including the statistical parametric maps (blue: hypoactivations; red: hyperactivations). Results are displayed at a *p* < 0.001, uncorrected threshold and neurologically displayed on axial slices using the Multi-image Analysis GUI as available at http://ric.uthscsa.edu/mango/mango.html.

Further investigations on the influence of emotion on cognition within peak regions of interests as based on FWE-corrected peak regions derived from the here identified emotion- (Neg>Neu trials) and cognition (IC>S) network revealed a significant trend within left amygdala, right insula and right precentral gyrus to show decreases of neural activation along for emotional primes with increasing cognitive demand. More specifically paired two samples *t*-tests indicated significant decreases of neural activation for (Neg>Neu) from congruent to incongruent condition within left amygdala, right insula, and right precentral gyrus (all *p* < 0.05). For the right insula the comparison between stars and incongruent condition likewise became significant (see Figure 4; additional graphics for the remaining regions that did not reach significance see Supplemental Information 2.2).

## Discussion

In this study, we investigated the influence of emotions on cognition in each corresponding network through the use of negative or neutral primes prior to a number Stroop task with increasing levels of cognitive demand. Our main behavioral findings demonstrated increased reaction time and reduced Stroop-task accuracy following negative primes and/or increasing cognitive demand. Neurally, the emotional primes consistently activated emotion-related brain regions (including amygdala, insula, and prefrontal brain regions) while the Stroop effect was associated with activations in areas linked to cognitive processing (including left inferior frontal junction/precentral gyrus, inferior/superior parietal lobe, and insula). And finally, the neural correlates representing the influence of emotion on cognition implementing variations in cognitive demand lead to decreases in neural activation in response to emotional stimuli (negative>neutral) along with increased cognitive demand within prefrontal cortex, amygdala, and insular cortex. Additionally, in trials with no preceding emotional prime (neutral>negative), significant increases along with increasing cognitive demand where observed. Overall we conclude that neural activation during a number Stroop task performance is increasingly disrupted by preceding negative images along with increasing cognitive demand.

### Behavioral effects following emotional primes and stroop trials with different cognitive load

In line with previous work, negative compared to neutral primes prior to a Stroop task affected task performance, which resulted in decreased task accuracy and increased reaction times (Gray et al., 2002; Mitchell et al., 2006; Blair et al., 2007; Padmala et al., 2011). Furthermore, more incorrect answers were given during incongruent trials compared to congruent (Etkin et al., 2006) or stars trials, while the latter two showed similar accuracy measurements. Participants were fastest in responding to congruent trials, followed by stars trials and eventually incongruent trials, which reflects the so-called Stroop effect (Stroop, 1935; Blair et al., 2007; Hart et al., 2010). We propose that in this study reading the number was a more automatized/faster process compared to counting the actual stimuli, thus resulting in shorter reaction times for congruent compared to stars trials. Interestingly, congruent and stars trials had equivalent numbers of correctly answered trials, although participants responded faster to congruent than to stars trials. We therefore conclude that the behavioral advantage of the congruent condition only affected reaction time (increased speed), but not accuracy. However, behavioral analysis failed to observe an interaction effect between emotional priming and task difficulty in accuracy and reaction times. In line with the dual competition model by Pessoa (2009) and supported by previous studies (Hart et al., 2010; Melcher et al., 2011; Padmala et al., 2011) the interference of negative primes on task performance was expected to augment with higher cognitive task load due to competing mechanisms. However, in line with previous evidence (e.g., Blair et al., 2007; Van Dillen et al., 2009), we did not observe a significant interaction effect in the present analysis.

### Neural basis of emotion–cognition interaction

Here, we demonstrate that the influence of emotion on cognition is neurally reflected within brain regions including prefrontal cortex, amygdala, and insular cortex. Furthermore, activation in these brain regions shows an attenuating trend when increasing cognitive demand. Likewise, neural activation in the left inferior frontal junction/precentral gyrus, as indicative of cognitive control, is increased during neutral prime trials compared to trials following a negative prime. Prefrontal brain regions, amygdala, and insula have all consistently been identified as relevant for emotion–cognition interactions or emotional conflict resolution (Gray et al., 2002; Beer et al., 2006; Etkin et al., 2006; Blair et al., 2007; Hart et al., 2010; Gu et al., 2013; Buhle et al., 2014). Likewise, similar areas are activated during tasks requiring cognitive reappraisal (an emotion regulation strategy in which the stimulus meaning is reinterpreted to downregulate the emotional valence; Ochsner et al., 2002, 2004; Ochsner and Gross, 2005). Thus, we suggest that the here presented emotional number Stroop task activates similar areas within the neural network that are required for deliberate cognitive reappraisal.

The *prefrontal cortex* can functionally and cytoarchitectonically be subdivided into distinct sub-regions, several of which are of relevance to affective processing, cognition, or both (Pessoa, 2008). Overall, prefrontal brain regions are commonly linked to attention, working memory, goal-directed behavior (e.g., cognitive control or decision making (Pessoa, 2008; Stokes et al., 2013; Leech and Sharp, 2014), and affective processing (Phan et al., 2002). From an evolutionary perspective, early research has suggested that the evolution of the human prefrontal cortex, particularly its expansion in volume, may reflect the development of more complex social behavior (Dunbar and Shultz, 2007). While such an interpretation may be too simplistic, still researchers commonly agree that the distinct parts of the prefrontal cortex are recruited by different high-level cognitive demands (Eickhoff et al., 2016). Based on animal and human studies, a functional and cytoarchitectonical subdivision of the medial prefrontal cortex may at least result in areas including the orbitofrontal cortex (BA11), ventral prefrontal cortex and prefrontal pole (BA10), and dorsomedial prefrontal cortex and frontal pole (BA9; Eickhoff et al., 2016). All of these areas are strongly interconnected and associated with various other circuitries of the brain, including the limbic network (Reid et al., 2016). Finally, particularly the right inferior frontal gyrus has been suggested to be a crucial hub during inhibitory processing and may consequently be an area affected in response control disorders (Aron et al., 2014).

Emotion processing is generally assigned to *medial prefrontal* brain regions, whereas the amygdala, anterior cingulate cortex, or insula are thought to possess a more distinct function within emotional tasks (Phan et al., 2002). The *amygdala* is one of the most traditionally viewed emotion and motivation processing center. With its relatively small structure, the amygdala nevertheless comprises a multitude of anatomical connections allowing many intricate functionalities (Janak and Tye, 2015). While abundant research has linked the amygdala to affective processing and particularly fear conditioning, strong evidence points toward a more integral role of the amygdala as a key node for valence processing during different aversive states, including fear, anxiety, or reward processing (Murray et al., 2014; Janak and Tye, 2015). Here we demonstrated that the amount of amygdala activation obtained during emotion–cognition interaction was highest during Stroop trials with lowest cognitive demand and decreases with increasing task difficulty. This finding is in line with Etkin et al. (2006) as well as Blair et al. (2007) that found decreases in neural activation within the amygdala during concurrent task with increasing cognitive demand. In line with previous suggestions (Etkin et al., 2006), it could be concluded that amygdala activation mirrors the amount of emotional conflict, rather than the resolution of such. Importantly the amygdala is strongly interconnected with areas of the prefrontal/orbitofrontal cortex (Davidson et al., 2000). This bi-directional connection allows regulatory processes important for mental well-being. For example, early deprivation or life stress may lead to disruption in amygdala-prefrontal coupling and patients with symptoms including anxiety, posttraumatic stress disorder, or heightened aggression oftentimes show structural and functional impairments within these circuitries (Gee et al., 2013).

The present task closely resembles two prior fMRI study designs (Blair et al., 2007; Hart et al., 2010). With the exception of adaptations that account for the age of the participants being tested (i.e., use of only negative stimuli and age-appropriate images), it may be considered a replication study. The present manuscript used an adapted version of an affective number Stroop task as implemented by Hart et al. (2010) and resulted in comparable findings. Reproducibility of scientific studies is crucial in order to inform about the robustness of an observed phenomenon (Martin and Clarke, 2017). Comparing our findings more closely to these two prior studies confirm the following main findings: (1) In line with both studies, negative primes slowed the participants' reaction time during the number Stroop task and are thus confirmed to interrupt goal-directed processing; (2) In line with Blair et al. (2007) increasing cognitive demand led to decreases in emotion-related brain regions (e.g., amygdala, insula, prefrontal cortex). Hart et al. (2010), observed the same trend however, no neural decreases in dorsolateral prefrontal areas during incongruent trials. Therefore, the authors concluded that high cognitive demand may override the attenuation effect in the prefrontal cortex. In contrast to Hart et al. (2010), we only observed decreases in neural activation with increasing cognitive demand. It remains to be investigated whether such a difference may be due to the number and characteristics of participants tested (*N* = 14; 5 males in Hart et al. (2010)/*N* = 30; 15 males in the present study) or are potentially due to the difference in stimuli choice and/or slightly longer presentation (an additional 500 ms) of the Stroop trial in our study. It is important to note that the slightly longer Stroop presentation rate was chosen due to the aim of consequently applying this task in younger participants.

The *insula* is a functionally heterogeneous brain region which is situated in the depth of the Sylvian fissure and may be divided into three sections: a dorsal anterior, a ventral anterior, and a posterior part (Nieuwenhuys, 2012; Uddin et al., 2014). The anterior insular cortex is, mostly bilaterally, connected to limbic and prefrontal brain regions (e.g., the amygdala), while the posterior part is more strongly interconnected with parietal, occipital and temporal parts of the brain (Kurth et al., 2010; Nieuwenhuys, 2012). The insula has shown to be activated during a wide range of functions, including auditory processing, vestibular and somatosensory functions, the perception of pain and temperature, viscerosensation, taste, olfactory processing, somatomotor control and motor plasticity, speech production, cognitive control, bodily awareness, as well as emotion processing (Nieuwenhuys, 2012). Importantly, according to research the anterior insula has a critical role during the regulation of social behavior, since its structure and function is altered in individuals with social disorders (including disruptive behavior disorder Sterzer et al., 2007; Raschle et al., 2015). Here our results are in line with findings assigning a critical role for the insula in emotion–cognition interactions (Hart et al., 2010; Shackman et al., 2011; Gu et al., 2013).

### Emotion–cognition integration in psychiatric disorders

An intact integration and healthy balance of competing emotion-cognition processing is crucial for our everyday functioning and an imbalance, as for example observed in individuals with emotion processing deficits, is linked to different mental health disorders (Monk, 2008). For example, faulty integration or regulation of emotion-cognition processes may result in heightened violence and aggression (Davidson et al., 2000). Therefore, it has been suggested that individuals with heightened aggression traits, as for example observed in children and adolescents with disruptive behavior disorders, may show impairments in these prefrontal circuitries responsible for successful emotion–cognition interaction and regulation. In fact, various structural and functional neuroimaging studies pinpoint areas of the limbic and prefrontal network to be disrupted in aggressive individuals (Raschle et al., 2015; Rogers and De Brito, 2016). Consequently, we conclude that future studies may implement the here presented design in order to further characterize aggressive youths and potentially impact individualized classification and treatment approaches in health and disease.

## Limitations

A potential caveat in the design of this study concerns the valence of the primes used. While we employed negative and neutral images only, positive primes have also been shown to disrupt task performance (Mitchell et al., 2006; Blair et al., 2007). Therefore, the addition of positive images should be considered in future studies aiming at characterizing the emotion–cognition interaction. However, due to practical challenges when conducting pediatric neuroimaging studies (particularly time constraints; for a discussion see for example Raschle et al., 2009) we decided that it is of importance to keep the task as short as possible and gain maximum power for the emotional condition chosen. Additionally, since the processing of negative affect is a particular problem in disruptive behavior disorders, a focus on this emotion made most sense. Secondly, we here used DAPS images as primes. This system was developed as an adaptation of the International Affective Picture System (IAPS, Lang et al., 2008) in order to be suitable for children and adolescents. The DAPS includes images from the IAPS series as well as additional stimulus material. We here only used images that were part of the IAPS and DAPS system, which can thus be considered suitable for evoking negative affect in both populations. However, IAPS images with the strongest negative affect were excluded within this process. Therefore, the images implemented here may have had a reduced impact on the young adults performing our task. Our decision to employ a child-friendly image system is due to our aim of testing our task for future use in clinical populations involving children and adolescents with disruptive behavior disorders. While effects of negative priming were observable in the young adults sample investigated here, we believe the same images may lead to stronger behavioral and neural effects in younger participants as investigated in the future. Finally, while we implemented an automatic stochastic schedule for optimal event presentation and null trials for jittering, the inter-trial intervals may gain from additional variance (i.e., variations in inter-trial intervals around for example 500–1,500 ms).

## Conclusion

Converging evidence points toward the importance of a balanced handling of both emotional and cognitive information in our everyday life. Here we present data that validates the usefulness of the emotional number Stroop task in fMRI settings aiming to assess the neural correlates of the influence of emotion on cognition. More specifically, we show an impact in behavior and the associated neural networks depending on emotional prime and cognitive demand. The respective influence of the emotion and cognition network in the brain may therefore be seen as a dynamic process which is modulated by the executive resources available. Moreover, emotion and cognition seem to be tightly related to each other, as indicated by shared neural networks involved in both of these processes. A failure to successfully integrate emotional and cognitive demands is characteristic to many psychiatric disorders. Future studies may thus further investigate the neural characteristics of children and adolescents that fail to successfully process emotional/cognitive demand, as for example seen in disruptive behavior disorders.

## Ethics statement

This study was carried out in accordance with the recommendations of the Ethikkommission der Nordwest- und Zentralschweiz. All subjects gave written informed consent in accordance with the Declaration of Helsinki. The protocol was approved by the Ethikkommission der Nordwest- und Zentralschweiz.

## Author contributions

Conception and design of the experiments: CS, PS, NR, FE, and WM. Data collection: LF, WM, and NR. Data analysis and interpretation: NR, CS, PS, LF, and WM. Drafting the paper: NR, CS, LF, and WM. Revision and final approval of the version to be published: NR, CS, PS, LF, WM, and FE.

### Conflict of interest statement

The authors declare that the research was conducted in the absence of any commercial or financial relationships that could be construed as a potential conflict of interest.
