# Supplementary material for: Investigating the Neural Correlates of Emotion–Cognition Interaction Using an Affective Stroop Task
Source: Front Psychol. 2017 Sep 1;8:1489. doi: 10.3389/fpsyg.2017.01489 (PMC5585191; doi:10.3389/fpsyg.2017.01489)

Supplementary Material

# The influence of emotion on cognition in prefrontal and limbic brain regions

**Nora Maria Raschle1*^, Lynn Valérie Fehlbaum1^, Willeke Martine Menks^1^, Felix Euler^1^, Philipp Sterzer^2^, Christina Stadler^1^**

*** Correspondence:** Corresponding Author: [nora.raschle@upkbs.ch](mailto:nora.raschle@upkbs.ch)

**^** indicates shared first authorship

# Supplementary Data

**1.1** **Negative and neutral images used in the emotional priming task.** All images are part of the DAPS (Cordon et al., 2013), mirroring the IAPS commonly used in adults (Lang, 2008). Numbers correspond to DAPS coding.

| 1300 | 2222 | 2400 | 3180 | 6510 | 9007 | 9530 |
| --- | --- | --- | --- | --- | --- | --- |
| 1410 | 2273 | 2487 | 3250 | 6830 | 9040 | 9570 |
| 1441 | 2274 | 2488 | 3500 | 6831 | 9050 | 9571 |
| 1500 | 2299 | 2506 | 3530 | 7002 | 9120 | 9600 |
| 1540 | 2302 | 2515 | 5201 | 7004 | 9140 | 9620 |
| 1620 | 2308 | 2518 | 5202 | 7081 | 9180 | 9622 |
| 1670 | 2314 | 2560 | 5390 | 7090 | 9181 | 9630 |
| 1850 | 2342 | 2580 | 5870 | 7130 | 9230 | 9910 |
| 1942 | 2382 | 2593 | 5940 | 7242 | 9250 | 9911 |
| 2018 | 2383 | 2594 | 6190 | 7440 | 9400 | 9912 |
| 2026 | 2384 | 2691 | 6210 | 7492 | 9420 | 9921 |
| 2032 | 2385 | 2791 | 6211 | 7509 | 9421 |  |
| 2036 | 2388 | 2800 | 6250 | 7512 | 9430 |  |
| 2037 | 2390 | 2870 | 6260 | 7595 | 9440 |  |
| 2053 | 2393 | 2900 | 6300 | 7820 | 9470 |  |
| 2058 | 2396 | 3030 | 6312 | 8230 | 9490 |  |
| 2205 | 2398 | 3160 | 6370 | 8480 | 9500 |  |

Cordon, I.M., Melinder, A.M., Goodman, G.S., and Edelstein, R.S. (2013). Children's and adults' memory for emotional pictures: examining age-related patterns using the Developmental Affective Photo System. *J Exp Child Psychol* 114(2)**,** 339-356. doi: 10.1016/j.jecp.2012.08.004.

Lang, P.J., Bradley, M.M., & Cuthbert, B.N. (2008). "International affective picture system (IAPS): Affective ratings of pictures and instruction manual. Technical Report A-8.". (Gainesville, FL.: University of Florida).

**1.2.** **Behavioral pilot study assessing in-scanner task**

A pilot study was run to validate the neuroimaging task behaviorally in 8 healthy adults (1 dataset was discarded due to incomplete data). Data was analyzed with regards to accuracy and reaction times with two separate 2 (emotion: Neg, Neu) by 3 (task: C, S, IC) repeated measures ANOVAs. Pilot data assessment indicated a significant emotion by cognition interaction on both accuracy measures (F(2,5)=9.224, *p*=.021) and reaction times (F(2,5)=14.451, *p*=.008). Moreover, a significant main effect of cognition was detected in accuracy measures (*F*(2,5)=182.542, *p*<0.001) and reaction times (*F*(1,6)=30.470, *p*=0.002).

|  |  | **Congruent** | **Stars** | **Incongruent** |
| --- | --- | --- | --- | --- |
|  |  | **[±SD]** | **[±SD]** | **[±SD]** |
| **Accuracy** | Negative prime | 50.0 [1.5] | 42.9 [1.07] | 46.0 [2.9] |
| **[raw scores]** | Neutral prime | 49.4 [1.7] | 40.6 [2.4] | 46.6 [3.36] |
|  |  |  |  |  |
| **Reaction Times** | Negative prime | 672.4 [82.7] | 684.9 [89.5] | 761.4 [113.3] |
| **[ms]** | Neutral prime | 663.7 [91.0] | 696.8 [102.0] | 784.0 [109.3] |
|  |  |  |  |  |
|  |  |  |  |  |

# Supplementary Figures and Tables

| **2.1** **Emotional valence rating** (N=30, raw scores). | | | |  |  | |
| --- | --- | --- | --- | --- | --- | --- |
|  | **Range** | **Mean** | **Standard Deviation** | | |  |
| Negative primes | 1.75 | -1.20 | 0.44 | | |  |
| Neutral primes | 2.13 | 0.66 | 0.46 | | |  |
|  |  |  |  | | |  |

## 2.2 Supplementary Figure 1. Additional graphs displaying follow-up investigations on the influence of emotion on cognition within regions of interests (right amygdala, left precentral gyrus).


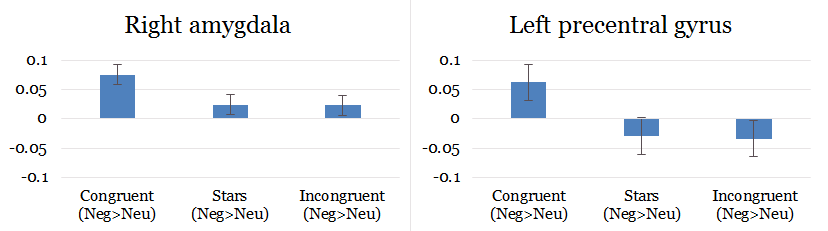

Supplement: Supplementary file 1 [file DataSheet1.DOC]
